# A Suicide Monitoring and Crisis Intervention Strategy Based on Knowledge Graph Technology for “Tree Hole” Microblog Users in China

**DOI:** 10.3389/fpsyg.2021.674481

**Published:** 2021-10-25

**Authors:** Bing Xiang Yang, Lin Xia, Lianzhong Liu, Wentao Nie, Qian Liu, Xin Yi Li, Meng Qin Ao, Xiao Qin Wang, Ya Dian Xie, Zhongchun Liu, Yi Jia Huang, Zhisheng Huang, Xuan Gong, Dan Luo

**Affiliations:** ^1^School of Health Sciences, Wuhan University, Wuhan, China; ^2^Department of Psychiatry, Renmin Hospital of Wuhan University, Wuhan, China; ^3^Population and Health Research Center, Wuhan University, Wuhan, China; ^4^Wuhan Mental Health Center, Wuhan, China; ^5^Teaching Office, Zhongnan Hospital of Wuhan University, Wuhan, China; ^6^Division of Mathematics and Computer Science, Faculty of Sciences, Vrije University Amsterdam, Amsterdam, Netherlands

**Keywords:** suicide, crisis intervention, knowledge graph, tree hole of Weibo, artificial intelligence, social media

## Abstract

“Zou Fan” is currently the largest “tree hole” on Weibo, where people having suicidal ideation often express their thoughts and use this channel to seek support. Therefore, early suicide monitoring and timely crisis intervention based on artificial intelligence technology are needed for this social media user group. This research was based on the knowledge graph technology, whereby “Tree Hole Intelligent Agent” (i.e., Artificial Intelligence Program) was used to identify “Zou Fan Tree Hole” users at high risk for suicide, and then, the “Tree Hole Action” carried out proactive suicide crisis intervention with them. The “Tree Hole Action” has temporarily prevented 3,629 potential suicides. The “Tree Hole Action” plays a significant role in suicide risk monitoring and crisis intervention for social media users and has been seen to have an important social impact.

## Introduction

Suicide is a global phenomenon and occurs at all stages of life. The data released by the World Health Organization [WHO] (2019) show that approximately 800,000 people die due to suicide worldwide each year, and 79% of victims were from low- and middle-income countries. Globally, suicide is the leading cause of death among people aged 15–29 years (World Health Organization [WHO], 2019), posing a significant threat to adolescents and young adults. The suicide rate of Chinese women in 2017 was 7.5 and that of men was 10.7 per 100,000 population (Chinese Center for Disease Control and Prevention, 2019a). Suicide is a tragedy for families, communities, and the entire country. It results in a loss of family productivity and income as well as an increase in the financial burden of the family (Bachmann, 2018). Suicide also has a long-term impact on the relatives and friends of the deceased and poses a mental burden on them (Chinese Center for Disease Control and Prevention, 2019b).

Although suicide is a serious and complex problem, it is possible to prevent suicide by adopting timely, low-cost, and evidence-based intervention methods (World Health Organization [WHO], 2019). Affected by stigma and social taboos, people with suicidal ideation rarely express their thoughts or seek help from families, friends, or professionals (Cheng et al., 2017). On the contrary, with the rapid development of Internet-based social networks in recent years, people express their feelings and opinions in virtual communities and platforms. For example, in China, Weibo has become the primary channel for social media users to express suicidal thoughts and seek support (Fu et al., 2013; Guan et al., 2015a). The term “tree hole” comes from the ancient fairy tale “King Donkey Ears,” and it was later circulated to refer to a place where one expresses his/her feelings (Lv, 2012). Driven by modern social media, Weibo has become a place where many “tree holes” exist. When a Weibo user committed suicide each time, the Weibo account became a “tree hole” for others experiencing emotional crises to express feelings in the comments field due to its more insidious characteristics (Yang et al., 2019). “Zou Fan Tree Hole” is the largest one on Sina Weibo and serves as an example. In 2012, after the suicide death of a young girl with the user name of “Zou Fan” on Sina Weibo, her Weibo received more than two million comments from 350,000 users. The majority of the comments were made during nighttime and focused on suicidal ideation (Huang et al., 2019b; Yang et al., 2019). The fact that many of these users who expressed suicidal thoughts in “Zou Fan Tree Hole” had suicidal behaviors later. Suicide monitoring and crisis intervention are needed for this group of social media users.

In recent years, the emergence of artificial intelligence (AI) technology has provided a new way to predict, monitor, and manage the suicidal behavior of social media users. The AI technology can generate risk algorithms that rely on big data to predict the outbreak of suicide and identify individuals or populations at risk (Fonseka et al., 2019). A study found that the risk classification accuracy of both AI and machine learning technology exceeded 90% (Bernert et al., 2020). Zhu (2019) used machine learning technology to create a suicidal ideation detection system on Weibo. The system can perform the real-time analysis of various Weibo content and can timely identify comments with suicidal ideation (Huang et al., 2014; Zhu, 2019). The well-known American social media “Facebook” also uses AI and machine learning technology to identify suicidal ideation posts or live videos (de Andrade et al., 2018). Besides, a series of suicide prevention and help-seeking apps and services, such as Radar, Woebot, and Voice Assistant Services, have been developed for users at risk of suicide (de Andrade et al., 2018).

For individuals at risk of suicide, prevention is a continuous process, including risk assessment and crisis intervention (Li, 2007). The AI technology has shown superior performance in suicide risk identification and monitoring and provides a basis for subsequent intervention (Vogel, 2018). The purpose of this study was to report on the “Tree Hole Action,” which uses knowledge graph technology to identify and classify the suicide risk of users in the “Zou Fan Tree Hole” site and to conduct suicide crisis intervention for those with high suicide risk at levels 6–10 (having a definite suicide plan or suicide in progress). This project effectively integrates AI and mental health services in a collaborative strategy using online and offline resources to provide practical guidance for suicide risk monitoring and crisis intervention for social media users.

## Materials and Methods

This study aimed to describe the development of a suicide monitoring and crisis intervention strategy named “Tree Hole Action” and to assess the effect of its implementation. The effect of implementation in this study refers to the reported number of messages at high suicide risk, the reported number of users with high suicide risk, the number of people rescued, and the internal structure and regional organization that has been formed. The whole study includes two phases: one is the establishment and development of “Tree Hole Action” and the other is the implementation of crisis intervention to prevent suicide.

### Phase I: Establishment and Development of “Tree Hole Action”

#### Suicide Risk Identification and Classification Based on Knowledge Graph Technology

Based on the Intelligent Agent system of the semantic data processing platform and combined with the knowledge graph technology, Huang Zhisheng developed the “Tree Hole Intelligent Agent” (Huang et al., 2019a). It comprises four processing modules, namely, data capture, data aggregation, data analysis, and report generation (Huang et al., 2019a). It monitors posted comments in the “Zou Fan Tree Hole” and performs intelligent analysis 24 h/day. It grabs the data every day from the “tree hole” website and extracts eight data attributes for each message, then uses a suicide knowledge graph and risk identification rule algorithm to analyze the data and generate “tree hole” monitoring reports. In the process of data analysis, natural language processing tools were used for word segmentation and syntactic parse. Meanwhile, based on the “Tree Hole Knowledge Graph” and risk identification rule algorithm, the messages of suicide risk level 6 and above were extracted. These risk identification rules adopt Definite Clause Grammars (DCG) Transformation Rules in the logical programming language Prolog. Based on the DCG rules, an extended description of knowledge graph reasoning ability is added, and domain knowledge is obtained from the knowledge graph to interpret each piece of message, thereby determining its suicide risk level (Huang et al., 2019a).

**FIGURE 1:**
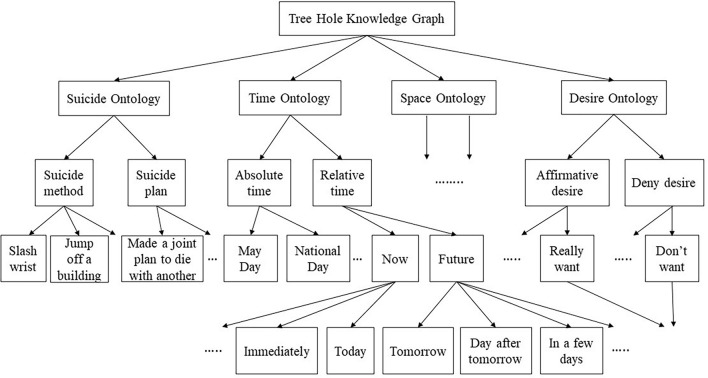
“Tree Hole Knowledge Graph” (Huang et al., 2019a).

The knowledge graph is a graph-based knowledge representation and organization method, which is a systematic, structured, and integrated domain-specific knowledge expressed in the form of semantic technology (Huang et al., 2019a; Li et al., 2020). To provide sufficient knowledge support for the “Tree Hole Intelligent Agent,” Huang Zhisheng constructed a “Tree Hole Knowledge Graph,” which is composed of suicide ontology, time ontology, space ontology, and desire ontology (Huang et al., 2019a). Suicide ontology focuses on suicide methods and suicide plans; time ontology covers the concepts of absolute and relative time; space ontology describes related concepts of spatial geography; and desire ontology is used to portray the subjective desires and related negative concepts of the people (Huang et al., 2019a). The “Tree Hole Knowledge Graph” provides various basic knowledge related to suicide and depression and is mainly used to permit the “Tree Hole Intelligent Agent” to judge the possibility of suicide contained in the information found in social media. The “Tree Hole Knowledge Graph” structure is shown in Figure 1.

Relying on the “Tree Hole Knowledge Graph,” the Agent can classify the suicide risk of “tree hole” users (according to the certainty of suicide methods and the urgency of time) and automatically identifies those users with high suicide risk levels of 6–10 (Huang et al., 2019a). The Agent then generates a “tree hole” monitoring report and sends it to the “Tree Hole Action” WeChat group. The suicide risk classification standards are as follows (Huang et al., 2019a): level 10 (suicide may be in progress), level 9 (suicide method has been determined and may occur soon), level 8 (suicide has been planned, and the suicide date is generally determined), level 7 (suicide method has been determined, and the suicide date is unknown), level 6 (suicide has been planned, and the suicide date is unknown), level 5 (expression of strong desire to commit suicide, and the suicide method is unknown), level 4 (suicidal desire has been expressed, and the specific method and plan are unknown), level 3 (intense survival pain, and no suicidal wishes expressed), level 2 (survival pain has been clearly expressed, and no suicidal wishes expressed), level 1 (survival pain is partially expressed, and no suicidal wishes expressed), and level 0 (no expression of survival pain noted).

#### Personnel Recruitment and Crisis Intervention Training for “Tree Hole Action”

##### Guideline of online suicide rescue

The “Tree Hole Action” has set up an online suicide prevention committee composed of crisis intervention experts, mental health and psychology nursing experts, and AI experts. Based on their rescue experiences and research-based scientific evidence on suicide crisis intervention, this committee has compiled a guideline for online suicide rescue so novice rescuers can better understand and guide the crisis intervention procedure. An outline of this guideline is shown in Table 1.

**TABLE 1:** Content outline of the guideline of online suicide prevention.

**Chapter number**	**Chapter title**	**Content of chapter**
One	Introduction	Purpose and significance of “Tree Hole Action”
Two	A brief introduction to “Tree Hole Action”	Technical means of “Tree Hole Action,” suicide risk classification standard, etc.
Three	Online suicide rescue standard process	Process of “Tree Hole Action”
Four	How to initiate a rescue	Interpretation of “tree hole” monitoring reports; select rescue targets from monitoring reports; identify false suicide information
Five	Method of establishing contact with the rescue target	How to contact rescue targets; what to do if targets do not respond; what should be done if rescue targets respond but refuse help; how to maintain communication with rescue targets, etc.
Six	Online suicide rescue team actions	Set up a rescue team; set up a caring team; contact rescue target’s family or friends; seek assistance from police; Weibo alarm; WeChat alarm; telephone alarm, etc.
Seven	Rescue team members and their positioning	Locate assistance to be provided to rescue targets; adaption process of rescue team volunteers after death of rescue target; whether there is a need for team operations, etc.
Eight	Rescue strategies for specific problems	Rescue strategies for emotional problems, domestic violence, school bullying, emotional problems related to homosexuality, financial debts, personality disorders, and serious disease issues
Nine	Matters needing attention	Matters needing attention in the rescue process
Ten	Concluding remarks	Further improvement of guideline of online suicide rescue
Appendix One	Official contact information of “Tree Hole Action”	Official Weibo address and email address of “Tree Hole Action”
Appendix Two	List of regional contact personnel of “Tree Hole Action”	Regional directors of 15 domestic regions as well as Europe, North America, and Asia Pacific
Appendix Three	News reports about “Tree Hole Action”	Domestic and foreign news reports about “Tree Hole Action”
Appendix Four	Teaching plan for third training course of “Tree Hole Action”	Suicide rescue training arrangements
Appendix Five	Special reminder classification in monitoring type of message reports	14 situations that require attention and how to deal with them

##### Volunteer recruitment and systematic training

To standardize the management of volunteer recruitment and training, the “Tree Hole Rescue Management Committee” developed regulations regarding volunteer management for “Tree Hole Action.” The members of “Tree Hole Action” joined the project voluntarily after they became aware of the project through academic seminars, news media reports, and the comments and recommendations of other team members. The regional leader of the “Tree Hole Action” conducts a preliminary review, followed by a final review by the management committee before a prospective volunteer is accepted as a team member. Volunteers must participate in two stages of online training on suicide prevention. The first stage of training focuses on how to use the guideline of online suicide rescue. This is followed by an online simple answer questions and case analysis evaluation, which examines how volunteers establish contact with rescue targets, assess suicide risk, identify depression symptoms, recognize ethical issues, and offer emergency responses in the rescue process. The total score is 100 (passing standard ≥ 60). The score range of volunteers is 37–100 (average score: 83.79), and the pass rate is 93.44%. Volunteers with scores below 60 will continue to take part in the next round of training until they pass the evaluation. The second stage of training includes 20 sessions of 1–1.5 h each, focusing on suicide prevention. The contents of this two-stage training are shown in Table 2. After the suicide rescue training, volunteers complete an online assessment with multiple-choice questions, which assessed the level of mastery of management regulations and situations of the volunteers requiring attention during a rescue. At this stage, volunteers can retake the assessment several times till they attain a score of 100. Only volunteers who have passed the training and evaluation can participate in “Tree Hole Action.”

**TABLE 2:** Pre-job training and suicide rescue training.

**Training type**	**Training session**	**Name of training course**
First stage of training: pre-job training	First	Basic process of “Tree Hole Action”
	Second	Basic process and matters needing attention of “tree hole” alarm; classification method and interpretation of “tree hole” information special reminder
	Third	Rescue strategies for certain types of “tree hole” users
	Fourth	Matters needing attention in “Tree Hole Action” and case analysis
Second stage of training: suicide rescue training	First	Basic knowledge of suicide rescue
	Second	Theories and methods of suicide prevention
	Third	Depression and suicide
	Fourth	Mental illness rehabilitation technology: theory and practice
	Fifth	Cognitive model of suicide: theory and application
	Sixth	Trust mechanism and rescue strategy of online suicide rescue
	Seventh	Application of psychological counseling technology in crisis intervention
	Eighth	Bioethics and death philosophy
	Ninth	Crisis intervention
	Tenth	Ethical issues of crisis intervention
	Eleventh	Psychological distress and suicide
	Twelfth	Cognitive impairment in patients with depression
	Thirteenth	Training of social psychology workers
	Fourteenth	Treatment of depression disorders
	Fifteenth	Principle and practice of “Tree Hole Action”
	Sixteenth	Hotline intervention process for high suicide risk
	Seventeenth	Six step model of suicide intervention
	Eighteenth	Bipolar depression and its intervention strategies
	Nineteenth	Substance dependence and suicide
	Twentieth	How to write a short paper on “Tree Hole Action”

There are currently 389 volunteers in “Tree Hole Action,” including more than 60 experts in psychiatry and psychology, 70 professionally trained psychological counselors, and other volunteers from all walks of life. Among them, men accounted for 33.4%, and women accounted for 66.6%. Volunteer members are between 18 and 69 years of age (*M* = 31.14, SD = 10.74), and 54.5% are below 30 years of age.

### Phase II: Implementation of Crisis Intervention to Prevent Suicide

#### Publish “Tree Hole” Monitoring Reports

The “Tree Hole Intelligent Agent” publishes “tree hole” monitoring reports daily on the “Tree Hole Action” WeChat group. These reports include “tree hole” messages, the date of the messages, suicide risk classification, gender, age, city, and Weibo links. If the suicide risk classification is level 6 and above, crisis intervention for suicide prevention is initiated.

#### Procedure of Crisis Intervention in “Tree Hole Action”

**FIGURE 2:**
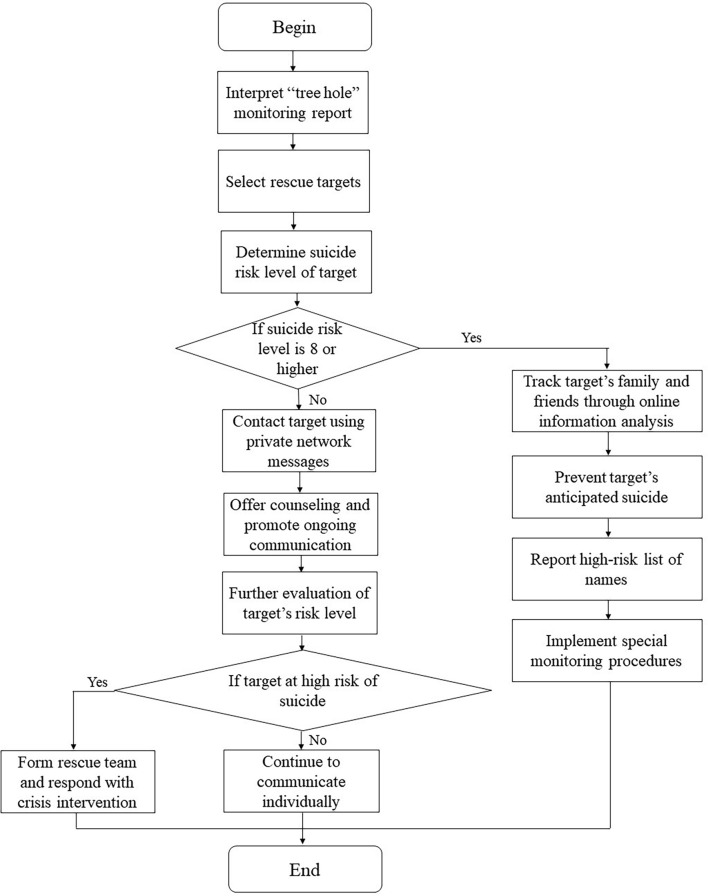
Procedure of “Tree Hole Action”.

Based on the interpretation of the “tree hole” monitoring reports, rescue team members select rescue targets and determine their risk level through personal communication *via* Sina Weibo. If the suicide risk of the rescue target is below level 8, the rescue team members will contact the target using private network messages, offer to counsel, and promote ongoing communication. If the target is at a risk level of 8 or higher, team members will try to track the family and friends of the targets through online information analysis, and suicide information will be shared with them to prevent the anticipated suicide of the target. Furthermore, all the targets at high risk (level 8 and above) will be added to a list of names, and special monitoring procedures will be implemented. The special monitoring procedures are for those users whose names are added to the high-risk list. “Tree Hole Intelligent Agent” will pay close attention to the messages of target users under “Zou Fan Tree Hole” and their Weibo dynamics. Date, time, Weibo ID, Weibo account, Weibo nickname, Message content, emotional symbol, Weibo nickname of the responder, and Weibo address of the responder will be extracted and analyzed (Huang et al., 2019a). Besides the AI analysis, a team member with professional backgrounds will contact the user actively through Weibo private messages and assess the suicidal ideation of the user. Once the person is found to have strong suicidal ideation or a suicide plan, a rescue team will be formed and respond with crisis intervention. Each rescue team is comprised of an expert in psychiatry in the geographical location where the target is located, a regional director, and other volunteers who have communicated with targets. Volunteers are responsible for collecting and sorting information about rescue targets, such as checking their previous microblogs, understanding their available support system, and determining their current problems. Psychological counselors or psychotherapists are responsible for offering counseling services, promoting ongoing communication, and implementing interventions in accordance with the six-step model of crisis intervention (James and Gilliland, 1982/2018). Regional directors are responsible for overall coordination, communicating with local resources, contacting the site, coordinating the alarm, and other related matters. If necessary, psychiatrists will be invited to answer questions about the use of medications. The process of “Tree Hole Action” is shown in Figure 2.

## Data Collection and Analysis

After participating in a suicide rescue, members of the rescue team each complete a “Tree Hole Action” personal workload form, and these data are submitted each quarter. The reports of rescue team members include the number of people at each rescue level (range: 1–5) each quarter. The rescue level is as follows: level 5 (sent messages to rescue targets, established a relationship of mutual trust, enabled the victim to actively express their emotions, and temporarily prevented the suicide; or directly participated in rescue operations to prevent collective suicides); level 4 (sent messages to rescue targets and established personal contacts; or contacted family members, work units, or the police to temporarily prevent the suicide); level 3 (sent messages to rescue targets, conducted multiple rounds of communication, and alleviated the suicidal mood of the victim); level 2 (sent messages to rescue targets and received responses, or joined a rescue group); and level 1 (sent messages to rescue targets, but no response was received).

## Results

### Summary of Monitoring Reports and Crisis Intervention for Suicide Prevention

From November 2018 to May 2020, the “Tree Hole Intelligent Agent” identified 5,766 high-risk messages of suicide among 3,236 “tree hole” users, and the high-risk messages reached 3,548 person-time. Among these users, there were 762 (23.55%) male users, 2,107 (65.11%) female users, and 367 (11.34%) users did not identify their gender. The number of messages from levels 6 to 9 was 1,193, 4,225, 48, and 300, respectively (Table 3).

**TABLE 3:** Users with high-risk messages identified by “Tree Hole Intelligent Agent” from November 2018 to May 2020.

**Suicide risk level**	**Messages**	**“Tree Hole” users (person-time)**	**“Tree Hole” users with one high-risk message (person-time)**	**“Tree Hole” users with more than two high-risk messages (person-time)**
Level 6	1,193	736	565	171
Level 7	4,225	2,517	1,863	654
Level 8	48	27	25	2
Level 9	300	268	246	22
Total	5,766	3,548*	2,699	849

*The 3,548 in the table refers to person-time, while the 3,236 in the article refers to the number of users with high suicide risk.

**FIGURE 3:**
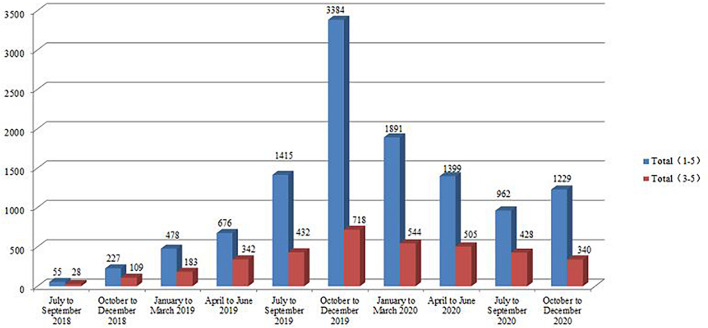
Total number of people rescued in levels 1–5 and 3–5 from July 2018 to December 2020.

During the crisis intervention from July 2018 to December 2020, the total number of people who received services from level 1 to level 5 was 11,716, while the number of people who received intervention from level 1 to level 5 was 5,283, 2,804, 1,969, 801, and 859, respectively (Table 4 and Figure 3). The “Tree Hole Action” prevented 3,629 potential suicides.

**TABLE 4:** Number of people rescued from level 1 to level 5.

**Year**	**Month**	**Level 1**	**Level 2**	**Level 3**	**Level 4**	**Level 5**	**Total 1–5**	**Total 3–5**
2018	July–September	15	12	16	5	7	55	28
	October–December	74	44	43	29	37	227	109
2019	January–March	222	73	66	69	48	478	183
	April–June	171	163	167	74	101	676	342
	July–September	687	296	213	108	111	1,415	432
	October–December	1,918	748	372	163	183	3,384	718
2020	January–March	967	380	328	116	100	1,891	544
	April–June	464	430	298	95	112	1,399	505
	July–September	232	302	244	74	110	962	428
	October–December	533	356	222	68	50	1,229	340
	Total	5,283	2,804	1,969	801	859	11,716	3,629

### Internal Structure and Regional Organization of “Tree Hole Action”

At present, “Tree Hole Action” has formed a systematic, standardized, and effectively managed internal organizational structure, including an organizational management committee, donation management committee, training committee, media working committee, ethics committee, and a technical committee. In addition, “Tree Hole Action” has established regional organizations in 15 domestic areas (e.g., Beijing, Zhejiang, Henan, Hubei, Guangdong, and Sichuan) and Europe, North America, and the Asia-Pacific region aiming to provide “tree hole” users at high suicide risk with timely, effective, and long-term intervention measures.

## Discussion

### Social Impact and Advantages of “Tree Hole Action”

The “Tree Hole Action” project, based on the knowledge graph technology and crisis intervention provided by the rescue team members, prevented 3,629 potential suicides from 2018 to 2020. Moreover, “Tree Hole Action” became a systematic, standardized, and effective organization to provide suicide prevention services. “Tree Hole Action” has made a significant social impact, has been a model for public crisis intervention, and was rated as the impact event of Sina Weibo in 2019. It has been reported by more than 100 media outlets globally, such as China News Service, Global Times, and Tokyo Shimbun of Japan. Moreover, “Tree Hole Action” has raised public awareness and understanding of suicide and attracted many people to volunteer for the project.

“Tree Hole Action” provides a normative and referenceable model for suicide risk monitoring and crisis intervention of social media users. The application of knowledge graph technology provides technical support for actively identifying Weibo users at risk of suicide. As mentioned, suicide risk identification and prediction are the basis of the project. Traditional methods of suicide prediction based on written questionnaires or scales are not adequately accurate, time-sensitive, and require the respondent to actively participate (Guan et al., 2015a; Cheng et al., 2017; Marks, 2019). The AI technology can make up for the shortcomings of traditional screening tools and improve suicide risk prediction accuracy (Marks, 2019). The knowledge graph can integrate all kinds of complex knowledge and data resources, i.e., a primary method for applying AI technology (Huang et al., 2019a). Huang Zhisheng applied the “Tree Hole Knowledge Graph” to the “Tree Hole Intelligent Agent” to judge the possibility of suicide in social media information, thereby identifying individuals with high suicide risk. The “Tree Hole Intelligent Agent” can accurately eliminate more than 99% of useless information, greatly reducing the workload of manual intervention and significantly improving the efficiency of the interpretation of “tree hole” information (Huang et al., 2019a). It can also carry out 24-h real-time monitoring of the “Zou Fan Tree Hole” and immediately send out suicide monitoring warnings, which may lead to successful suicide crisis intervention.

Meanwhile, the “Tree Hole Intelligent Agent” has high reliability and accuracy in suicide monitoring warnings. In the early stage, to evaluate the reliability of the “Tree Hole Intelligent Agent” in suicide risk classification, we randomly extracted monitoring reports during 16 days for analysis, which included a total of 21,356 “tree hole” messages and 163 messages with level 6 suicide risk or higher. By analyzing and evaluating these 163 messages, we found that the average correct rate of early warning information sent by the “Tree Hole Intelligent Agent” was 82% (Huang et al., 2019a). The suicide monitoring model constructed in this project based on the knowledge graph technology has shown great potential in identifying and monitoring the suicide risk of social media users. This innovative approach clarifies its value in monitoring large-scale populations (Guan et al., 2015b). At the same time, the current system still misses some information on high suicide risk, so it is necessary to improve and optimize the suicide risk identification algorithm to obtain more accurate warning results (Huang et al., 2019a).

“Tree Hole Action” integrates online and offline interventions, which intervene in suicide prevention more actively, continuously, and timely than other suicide crisis intervention methods. The crisis intervention hotline functioned as an important channel for many people with suicidal ideation to seek help and obtain support (Shaffer et al., 1988). Some researchers found that the crisis intervention hotline helped reduce the suicide risk of those with a high risk of suicide (Wang et al., 2011; Pang et al., 2015). However, the hotline worked passively for suicide prevention, in that it could not actively identify the suicide intention. Recently, the trend of more and more Internet social media users willing to express their thoughts online has provided a need for suicide crisis intervention based on social networks. It is a fruitful addition to the existing intervention methods that rely on the social network information of users for early identification and intervention of suicide risk and turn passive intervention into proactive intervention. In addition to “Tree Hole Action,” Zhu Tingshao used a machine learning model to identify Weibo users with suicidal thoughts and behaviors and offered them timely and effective interventions *via* direct message, providing information and emotional support (Liu et al., 2019; Zhu, 2019). For this kind of intervention, once individuals at high risk of suicide do not respond online, it will be difficult to stop their suicidal behavior. That is why “Tree Hole Action” set up an offline work team for continuing intervention, and it has shown success in rescue efforts. This professionally organized and social media-based suicide crisis intervention method could optimize the advantages of team members with diverse backgrounds and could improve the success rate of intervention. However, it is difficult to identify whether users with high suicide risk actually commit suicide subsequently. We can only use the existing data to show that “Tree Hole Action” prevented 3,629 potential suicides. Although the evaluation of the effect of Internet-based suicide crisis intervention has certain limitations, “Tree Hole Action” can achieve preventive effects and can enrich current suicide prevention strategies. The program is worthy of the long-term implementation and evaluation.

“Tree Hole Action” has a stable organizational structure, standardized rescue procedures, and systematic volunteer training, ensuring effective suicide crisis intervention. The various internal committees of “Tree Hole Action” have clear and reasonable duties, promoting suicide prevention. The rescue team conducted suicide intervention according to the guidelines and achieved good results, which confirmed that the procedures have good feasibility and provide appropriate guidance in the actual rescue process. As a non-profit organization, “Tree Hole Action” recruited many volunteers with great demand to effectively increase their skills and literacy. The systematic volunteer training program formed by “Tree Hole Action” functions well in training and providing qualified human resources for suicide rescue.

### Difficulties and Challenges Faced by “Tree Hole Action”

At the same time, “Tree Hole Action” has also faced challenges. First, the sustained development of “Tree Hole Action” demands careful consideration. Currently, the rescue team relies on individual volunteer applicants, and the consistency and stability of the volunteer team needs to be further enhanced. Second, “Tree Hole Action” mobilized social forces, including some non-professional volunteers in the rescue team. “Tree Hole Action” provided more than 20 h of training and continuous supervision for non-professionals, and the emergency rescue activities were carried out in small groups under the guidance of professionals. However, compared with other resources such as a psychological assistance hotline, “Tree Hole Action” is not a treatment activity that occurs within a professional institution, so its approach and purpose may still be questioned.

Meanwhile, “Tree Hole Action” is mainly carried out through the network, and it is difficult to track and supervise whether the behavior of an individual can be standardized. Third, the privacy protection of social media users and research involving ethical issues also deserves attention. When a Weibo private message is sent to a user at risk of suicide, but the user does not reply, there is a need to conduct a further evaluation based on the Weibo dynamics of the user. If there is sufficient evidence that the user is at high risk of suicide, the police are asked to intervene, which may cause controversy as to whether these actions interfere with the right of the user to die. Finally, the integration of online rescue and offline resources needs to be further improved, which is also an area for future research.

## Limitations

There are several limitations to this study. First, “Tree Hole Action” began to evaluate the effectiveness of the two-stage training program for volunteers in 2020, focusing on knowledge and skills. At present, there has been no assessment of the attitudes and satisfaction of volunteers with training courses. A comprehensive evaluation is planned by the current researchers. Second, the target population of “Tree Hole Action” is social media users, which means that individuals who have suicidal ideation but are not accustomed to expressing suicidal thoughts on social media can be difficult to identify and receive timely suicide crisis intervention. Third, the proportion of occurrences of suicide deaths and suicide attempts among individuals at different levels of suicide risk have not been analyzed. Future studies need to focus on the reliability and validity of the “Tree Hole Intelligent Agent” which have not been tested comprehensively in this study.

## Conclusion

“Tree Hole Action” is a successful example of using AI in coordination with mental health services. It effectively integrates AI technology, mental health professional resources, and social forces and plays a specific role in suicide risk monitoring and crisis intervention for social media users. However, the accumulated experience is also limited, and there is a need to further enrich and improve the relevant norms of online suicide rescue and prevention practices in the future.

## Data Availability Statement

The original contributions presented in the study are included in the article/supplementary material, further inquiries can be directed to the corresponding authors.

## Author Contributions

BXY, LX, LL, and WN completed the manuscript draft. XYL, MQA, and YDX did the literature review. QL, XQW, and ZL checked the data. ZH, XG, and DL contributed to the in-depth revisions of the manuscript. All authors contributed to and approved the final manuscript.

## Conflict of Interest

The authors declare that the research was conducted in the absence of any commercial or financial relationships that could be construed as a potential conflict of interest.

## Publisher’s Note

All claims expressed in this article are solely those of the authors and do not necessarily represent those of their affiliated organizations, or those of the publisher, the editors and the reviewers. Any product that may be evaluated in this article, or claim that may be made by its manufacturer, is not guaranteed or endorsed by the publisher.
